# Plasma Membrane Intrinsic Proteins *SlPIP2;1, SlPIP2;7* and *SlPIP2;5* Conferring Enhanced Drought Stress Tolerance in Tomato

**DOI:** 10.1038/srep31814

**Published:** 2016-08-22

**Authors:** Ren Li, Jinfang Wang, Shuangtao Li, Lei Zhang, Chuandong Qi, Sarah Weeda, Bing Zhao, Shuxin Ren, Yang-Dong Guo

**Affiliations:** 1 College of Horticulture, China Agricultural University, 100193 Beijing, China; 2 Institute of Vegetables and Flowers, Chinese Academy of Agricultural Sciences, 100081 Beijing, China; 3 School of Agriculture, Virginia State University, PO Box 9061, Petersburg, VA 23806, USA

## Abstract

The function of aquaporin (AQP) protein in transporting water is crucial for plants to survive in drought stress. With 47 homologues in tomato (*Solanum lycopersicum*) were reported, but the individual and integrated functions of aquaporins involved in drought response remains unclear. Here, three plasma membrane intrinsic protein genes, *SlPIP2;1, SlPIP2;7* and *SlPIP2;5,* were identified as candidate aquaporins genes because of highly expressed in tomato roots. Assay on expression in *Xenopus oocytes* demonstrated that SlPIP2s protein displayed water channel activity and facilitated water transport into the cells. With real-time PCR and *in situ* hybridization analysis, SlPIP2s were considered to be involved in response to drought treatment. To test its function, transgenic Arabidopsis and tomato lines overexpressing *SlPIP2;1, SlPIP2;7* or *SlPIP2;5* were generated. Compared with wild type, the over-expression of *SlPIP2;1, SlPIP2;7* or *SlPIP2;5* transgenic Arabidopsis and tomato plants all showed significantly higher hydraulic conductivity levels and survival rates under both normal and drought conditions. Taken together, this study concludes that aquaporins (*SlPIP2;1, SlPIP2;7* and *SlPIP2;5*) contribute substantially to root water uptake in tomato plants through improving plant water content and maintaining osmotic balance.

Water shortage is considered to be one of the most severe agricultural problems affecting plant growth^1^. In recent years, more attention has been paid to the possible role played by aquaporins in root water status and its relevance for defense mechanisms of plants against stress conditions.

Previous studies have shown that there are three pathways to transport water: the apoplastic, symplastic and transcellular pathways^2^. Aquaporins (AQPs), which play a regulatory role in cellular water transport^3^,^4^,^5^,^6^, also called membrane protein family MIP (major intrinsic protein)^7^,^8^,^9^. There are 47 aquaporin genes in the tomato genome which can be divided into five different subfamilies based on subcellular localization and sequence homology: plasma membrane intrinsic proteins (PIPs), tonoplast intrinsic proteins (TIPs), NOD26-like MIPs or (NIPs), small basic intrinsic proteins (SIPs)^10^,^11^ and the uncharacterized X intrinsic proteins (XIPs)^12^. Among PIPs, water channel activity is exhibited particularly by members of the PIP2 subgroup^13^. AQPs are considered the main channels for the transport of water along with small neutral solutes and CO_2_, through the plant cell membrane^9^,^14^.

Hydraulic regulation determines the interplay between water potential gradients and waterflow intensity throughout the whole plant^15^. Although osmotic hydraulic conductivity (Lp_r_) decreases upon root exposure to drought^16^, the root hydraulic conductivity of trembling aspen shown an increase under mild stress (exposing roots to a high humidity environment for 17 h)^17^. As previously indicated, Lp_r_ behavior is regulated partially by aquaporin function, specifically by PIPs: In *Melaleuca argentea*, the partial root-zone drying (PRD) can induce rapid changes in Lp_r_ and aquaporin expression in roots^18^. The Arabidopsis knockout mutants of PIP2;2 shown a lower hydraulic conductivity of root cortex cell compared with the wild type^19^. Overexpression of *LeAQP2* gene in root can enhanced hydraulic conductance which induced by arbuscular mycorrhizal fungi^20^. Zhou *et al*. found that the overexpression of GmPIP1;6 transgenic soybean plants did not have higher root hydraulic conductance (Lo) under normal conditions, but were able to maintain Lounder saline conditions compared to WT which decreased Lo^21^.

The importance of aquaporins in the responses of plants to environmental stresses has been implicated by the observation that the expression of aquaporin isoforms is differentially modulated by various environmental stresses, including high salinity, drought, and cold, in various plant species^22^. Yue *et al*. demonstrated that the expression of *CsPIPs* was induced by drought and remained relatively high after rehydration in leaves^23^. Ding *et al*. found that overexpression of a lily PIP1 gene in tobacco could greatly increase osmotic water permeability of leaf protoplasts and water conductivity of leaf cells^24^. Commonly a decrease in abundance of PIP2 proteins under drought conditions has been observed; although, in contrast, an accumulation of PIP1 proteins has been found^24^,^25^.

During the last several years, attempts to understand the roles of aquaporins in response to environmental stresses have prompted the analysis of transgenic plants and loss-of-function mutant lines^22^,^26^. A number of studies exist in which the expression of particular PIP or TIP isoforms is altered through overexpression or knock out of the respective gene^26^,^27^,^28^,^29^,^30^,^31^,^32^,^33^. Overexpression of BnPIP1 in transgenic tobacco plants resulted in an increased tolerance to water stress^34^. Transgenic rice plants overexpressing aquaporin RWC3 were more tolerant to drought stress and exhibited higher Lpr compared with non-transformed control plants^35^. Constitutive expression of SlTIP2;2 increased the osmotic water permeability of the cell and whole-plant transpiration in tomato^36^. Arabidopsis mutants lacking both TIP1;1 andTIP1;2 showed a minor increase in anthocyanin content, and a reduction in catalase activity, but showed no changes in water status^26^. Despite the increasing number of reports demonstrating the role of aquaporins in plant responses to environmental stresses, the function of each individual aquaporin isoform and the integrated function of aquaporins in response to various environmental stresses remain unclear.

Previous studies appear to select TIP as candidates for the improvement of plant abiotic stress tolerance in tomato on the basis of educated guesses^36^. In contrast, we selected three PIP type aquaporins (*SlPIP2;1, SlPIP2;7* and *SlPIP2;5*) as potential candidates based on their transcript abundance in root tissue. To better understand the role of three *SlPIP*2 genes in response to drought stress, overexpression vectors of *SlPIP2;1, SlPIP2;7* or *SlPIP2;5* were introduced into both Arabidopsisand tomato plants. Transgenic experiments indicated that *SlPIP2s* increase plants tolerance to drought stress by improving plant water content and maintaining osmotic balance. Thus, we supported the hypothesis that the constitutive expression of *SlPIP2;1, SlPIP2;7* and *SlPIP2;5* might transition plants from regular growth behavior to drought-tolerant growth behavior.

## Materials and Methods

### Plant Materials and Growth Conditions

Tomato (*Solanum lycopersicum*, cv. Micro Tom) seeds were surface sterilized with 75% (v/v) ethanol for 30 s and 2% (v/v) NaClO for 15 min, followed by washing five times with sterile water. The sterilized seeds were germinated on half-strength Murashige and Skoog (1/2 MS) medium in a growth chamber (16 h light/8 h dark, 24 °C/18 °C). After one month leaf, stem, flower and root tissues were harvested, frozen with liquid nitrogen, and preserved at −80 °C. *Arabidopsis thaliana* ecotype Columbia was grown on half-strength MS medium at 23 °C under a long day condition for 4-5 days after sterilized. After germination, seedlings were transferred to pots containing a 2:1:1 mixture of vermiculite, peat moss, and perlite and grown at 23 ± 2 °C under 12-h-light/12-h-dark cycle with a light intensity of 300 μEm^–2^s^–1^.

### Analysis of gene expression in different tissue and isolation of SlPIP2; 1, SlPIP2;7 and SlPIP2;5 cDNA

The open reading frames (ORFs) of 11 PIP and 9 TIP tomato aquaporin sequences were searched by using sol genomic network (http://solgenomics.net). RNA was isolated from tomato leaf, stem and root tissues using the Quick RNA Isolation Kit (Hua Yueyang Biotechnology, Beijing) following the manufacturer’s instructions. Quantitative real-time PCR (qRT-PCR) was performed in triplicate with an ABI Prism 7500 system using the SYBR Green PCR Master Mix kit (Applied Biosystems), according to the manufacturer’s instructions. A tomato actin gene (GenBank: GQ339765) was used as a standard control. Sequences of the primers used are available in Table S2. Gene expression data were analyzed by 7500 2.0 software (ABI) using −2^ΔΔCT^ method^37^. The expression of 20 aquaporin genes in different tissue was clustered in a heatmap using cluster 3.0 software.

### Sequence analysis

Nucleotide and amino acid sequences were analyzed using DNAman (DNAman Inc). The NCBI program (http://blast.ncbi.nlm.nih.gov/Blast.cgi) program was used for sequence alignment. Phylogenetic analysis was employed to investigate the evolutionary relationships among the aquaporins. A minimum evolution tree was generated in MEGA 4.0.

### Subcellular localization of SlPIP2;1, SlPIP2;7, and SlPIP2;5 proteins

To generate SlPIP2;1, SlPIP2;7, and SlPIP2;5 fused with GFP at the C-terminal, *SlPIP2;1, SlPIP2;7* and *SlPIP2;5* cDNAs were amplified by PCR using Taq DNA polymerase. The full-length open reading frame of *SlPIP2;1, SlPIP2;7* and *SlPIP2;5* was fused upstream of the GFP gene and put under the control of the constitutive 35S promoter in the pCAMBIA1302 expression vector to construct 35S:: SlPIP2;1, 35S:: SlPIP2;7 and 35S:: SlPIP2;5 fusion proteins. The specific primers used were: SlPIP2;1 F (5′-GGAAGATCTGATGTCGAAGGACGTGATT-3′); SlPIP2;1 R (5′-ACTAGTCAGATCTACCATGTTGGTT-3′); SlPIP2;7 F (5′-GGAAGATCTGATGGCAAAGATTATGT-3′); SlPIP2;7 R (5′-ACTAGTATTGGTGGCGTTGCTGCGG-3′); SlPIP2;5 F (5′-GGAAGATCTGATGACTAAAGAAGT-3′); SlPIP2;5 R (5′-ACTAGTCAGATCTACCATGGCAGTGC-3′).

The PCR product obtained was digested with the relevant restriction endonucleases (*Spe* I and *Bgl*II), ligated with the pCAMBIA1302 plasmid, and cut with the corresponding enzyme to create a recombinant plasmid for expressing the fusion protein. Recombinant constructs were transformed into living onion epidermal cells by biolistic bombardment (Model PDS-1000/He).

To induce plasmolysis, cells were incubated with0.8 M mannitol for 3–5 min. The subcellular location of *SlPIP2;1, SlPIP2;7*, and *SlPIP2;5* was detected by monitoring the transient expression of GFP in onion epidermal cells with a confocal microscope.

### *In situ* hybridization

Tissue preparation and *in situ* hybridization was performed as described by Regan *et al*.^38^. Digoxigenin-labelled antisense and sense probes for three AQPs (*SlPIP2;1, SlPIP2;7* and *SlPIP2;5*) were generated with the DIG RNA labeling kit as recommended by the manufacturer (Roche). Primers for cRNA probes were designed in the 3′ UTR cDNA region of candidate genes; an 18S ribosome cDNA was used as positive control (Table S4). Pieces (5 mm diameter) of young tomato root were fixed in 4% paraformaldehyde. After embedding in paraplast, 10- to 12-mm sections were transversally cut and affixed to microscope slides. The sections were deparaffinized and dehydrated with successive ethanol/water steps. Hybridization signals were detected with NBT, BCIP (Roche) and observed with a microscop^38^. Three independent batches of plants were analyzed.

### *In vitro* synthesis, oocyte preparation, cRNA injection, and Pf Measurements

The cDNAs of *SlPIP2;1, SlPIP2;7* and *SlPIP2;5* were subcloned into pCS107 vector using the restriction sites *Xho*l I and *Eco*R I. Capped cRNA transcripts were synthesized *in vitro* with mMACHINE SP6 Kit (Ambion) with Xba I linearized vector. *Xenopus laevis* oocytes of stages V and VI were isolated and defolliculated by digestion at room temperature for 1 h with 2 mg/ml collagenase A (Sigma) in ND96 solution (96 mM NaCl, 2 mM KCl, 1 mM CaCl_2_, 1 mM MgCl_2_and 5 mM Hepes-NaOH, pH 7.4, 220 mosm/kg). A 50 nl volume of *in vitro* transcripts (50 ng) of the target gene, using the same volume of distilled water as negative control, was injected into the oocytes. Non-injected oocytes were also analyzed as a negative control. The oocytes were incubated at 18 °C for 48 h in ND96 solution supplemented with 10 μg/ml penicillin and 10 μg/ml streptomycin (Fig. S1). To measure the osmotic water permeability coefficient (Pf), a single oocyte was transferred to 5-fold diluted ND96 solution. Oocyte volume (V) was calculated from the measured area of each oocyte. The osmotic permeability coefficient (Pf) was calculated for the first 5 min using the formula Pf = V0[d(V0/V)/dt]/[S0 × VW(Osmin-Osmout)], with an initial volume (V0) of 9 × 10^−4^ cm^3^, an initial oocyte surface area (S0) of 0.045 cm^2^, and a molar volume of water (V/V) of 18 cm^3^/mol^39^.

### Pf inhibition assays

For pH inhibition experiments, the oocyte internal or external pH was acidified by pre-incubating oocytes for 15 min in 50 mM sodium acetate (for internal pH modification) or NaCl (for only external pH modification), 20 mM MES pH 6.0 and mannitol until the desired osmolality was achieved (200 mOsmol/kg H_2_O). In order to reach pH 7.5, MES was replaced by HEPES in the solution described above. The swelling response was performed by transferring the oocyte to the same solution diluted 5-fold with distilled water. To test mercury inhibition, a stock solution of 100 μM HgCl_2_ was freshly prepared and oocytes were pre-incubated for 2 min in the presence of HgCl_2_ at a final concentration of 100 μM HgCl_2_. This concentration was maintained during the experiments.

### Measurement of Lp_r_ in a Pressurized Chamber

The root system of a freshly *Arabidopsis* plant was inserted into a pressure chamber filled with hydroponic culture medium as described^19^. Excised *Arabidopsis* roots were subjected to a pretreatment at 320 kPa for 15 min to attain equilibration, and Jv was measured successively at 320, 240 and 160 kPa for 5 min as described by Sutka^15^. The hydrostatic water conductivity of an individual root system (Lp_r_; ms^−1^Mpa^−1^) was calculated from the following equation using the sap flow Jv (P) relationship determined at three P values: Lp_r_ = J v/(Sr × P), Sr was integrated from total root length and diameter repartition. The excised tomato roots were subjected to a pretreatment at 600 kPa for 15 min to attain equilibration, and Jv was measured successively at 600, 500 and 400 kPa for 5 min, and Lp_r_ calculated as described above.

### Arabidopsis and tomato Transformation

To construct the overexpression vector, the ORF sequence of SlPIP2s were amplified using a pair of primers with *Xba*I and *BamH*I sites (Table S5), and the resulting PCR product was digested with *Xba*I and *BamH*I and then cloned into the pBI121 vector. Transformation of Arabidopsis was performed according to the flower dipping method^40^. The overexpression plasmids were introduced into Agrobacterium LBA4404 and transformed into tomato by Agrobacterium mediated transformation^41^.

### Transgenic plants seed germination and seedling growth assays under drought conditions

Arabidopsis seeds from individual plants were harvested on the same day and used for germination and seedling growth assays. Germination assays were carried out on three replicates of 20–30 seeds. Seeds were sown on 1/2 MS medium and placed at 4 °C for 2 days in the dark and then transferred to normal growth conditions. To determine the effect of dehydration on germination, the medium was supplemented with PEG6000^42^. To determine the effect of dehydration on seedling growth, both Arabidopsis and tomato seeds were grown on MS medium. Seven-day-old seedlings were planted in sieve-like rectangular plates (3 cm deep) filled with soil mixture and well watered. Tomato seedlings were cultured in a growth chamber (16 h light/8 h dark, 24 °C/18 °C) without watering for 15 d while Arabidopsis seedling were culturein another growth chamber (16 h light/8 h dark, 22 °C/16 °C, 70% relative humidity) without watering for 10 d.

### Quantitative RT-PCR analysis in root under abiotic stresses

The expression of *SlPIP2;1, SlPIP2;7* and *SlPIP2;5* genes in various tomato tissue under normal and abiotic stress conditions was analyzed by real-time quantitative reverse transcriptase using the fluorescent intercalating dye SYBRGreen in a detection system. A tomato actin gene (GenBank: GQ339765) was used as a standard control in the RT-PCR reactions. Time X is any treated time point (1, 3, 6, 12, 24, 48 and 72 h) and Time 0 represents the untreated time (0 h).

### Measurement of RWC, MDA Content

For the RWC assay in tomato, 4 week-old tomato plants were deprived of water for 15 d. Tomato leaves were sampled from WT and transgenic lines under drought stress for 15d to detect RWC. Fresh weight (FW) was immediately recorded; leaves were then soaked for 8 h in distilled water at room temperature in the dark, and the turgid weight (TW) was recorded. After drying for 24 h at 80 °C total dry weight (DW) was recorded. RWC was calculated as follows: RWC (%) = [(FW − DW)/ (TW − DW)]*100. MDA content was determined by the thiobarbituric acid (TBA)-based colorimetric method as described by Heath and Packer^43^.

### Abiotic stress tolerance assays

Drought tolerance assays were performed on seedlings. Tomato seeds were initially grown on MS medium. The 30-day-old seedlings were transplanted to plates (3 cm deep) filled containing liquid MS medium with 10% PEG6000. For salt tolerance assays, seedlings were transferred to plates containing liquid MS medium and 150 mM NaCl.

### Statistical analysis

All data were analyzed by one-way ANOVA and using Duncan’s multiple range tests (P < 0.05). The experiment had a completely randomized design. All values reported in this study were the means of three replicates.

## Results

### Isolation and sequence analysis of three SlPIPs genes from Micro Tom

**Figure 1:**
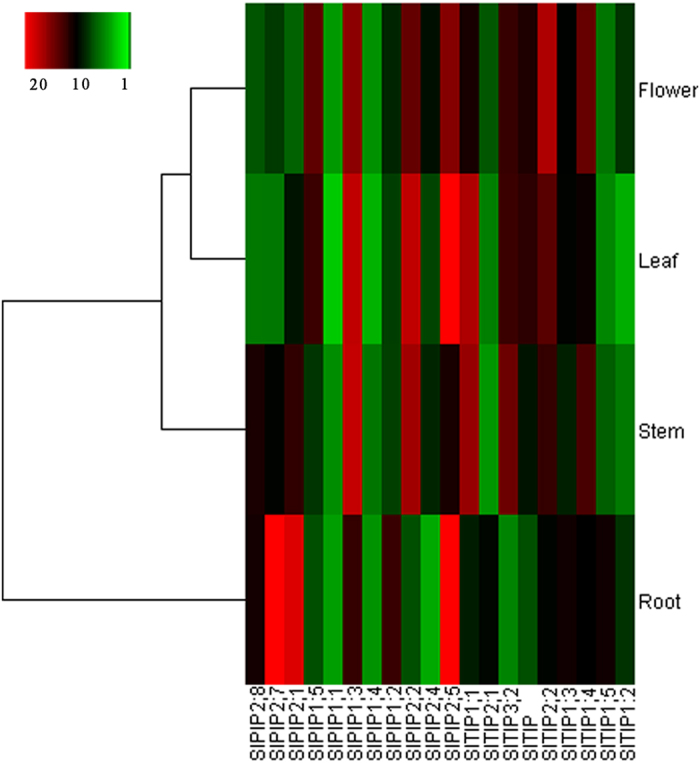
Heatmap of PIPs and TIPs expression in root, stem, flower, and root tissues of tomato plants. SlPIP2s transcript abundance in leaves, roots, stems and flowers was analyzed using qRT-PCR.The expression values were calculated using the 2^–Δt^ method and the 18S housekeeping gene, and the average log2 values of three replicates were used to generate a heat map in cluster 3.0 software. Green represents low expression, and red denotes high expression. (For interpretation of the references to color in this figure legend, the reader is referred to the web version of this article).

Using tomato genomic data(http://solgenomics.net/), we selected eleven PIP and nine TIP tomato aquaporin genes. Information including gene names, accession numbers, and the length of deduced polypeptides is summarized in Table S1. To evaluate the expression of candidate tomato aquaporin genes in different tissues, total RNA was prepared from various tissues (leaves, stems, flowers and roots) of 4-week-old tomato seedlings. In Fig. 1 the expression ratio of tomato aquaporin genes tested can been seen as mean values of the three biological replicates. Quantitative real-time PCR analysis revealed that eight aquaporins had higher expression levels in roots relative to other tissues. Among these genes, *SlPIP2;1, SlPIP2;7* and *SlPIP2;5*, showed significantly higher levels than other genes (Fig. 1, Table S3).The sequence information for SlPIP2;1, SlPIP2;7 and SlPIP2;5 has previously been reported by Reuscher *et al*. who used the names SlPIP2;8, SlPIP2;4 and SlPIP2;11, respectively.

**Figure 2:**
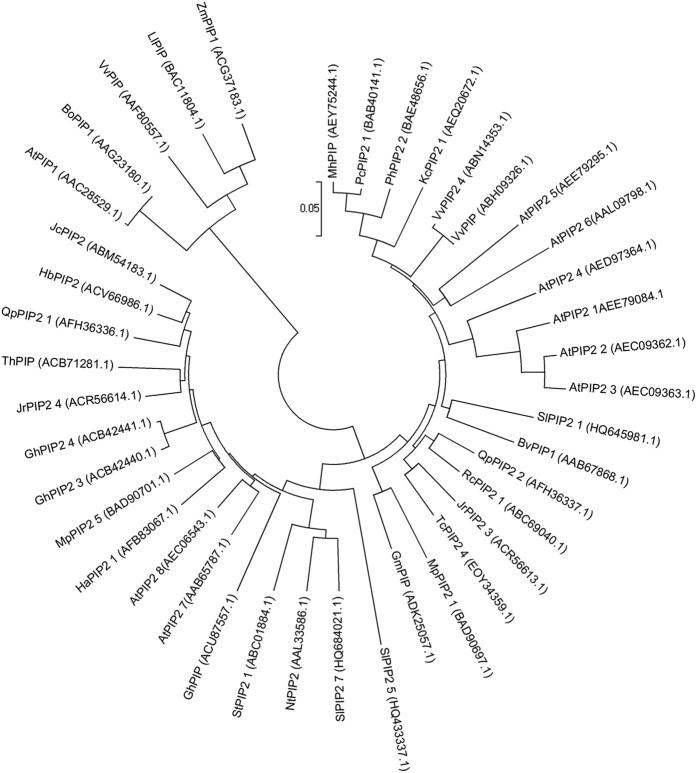
Phylogenetic relationship of *SlPIP2;1, SlPIP2;7* and *SlPIP2;5* with PIP proteins. The predicted amino acid sequences of the three SlPIP2s and their corresponding sequences from other species were aligned using the ClustalW2 sequence alignment program. The phylogenetic tree was constructed using MEGA6 software.

A comparison of the deduced polypeptides and a phylogenetic analysis of the SlPIP2;1, SlPIP2;7 and SlPIP2;5 with their counterparts from *Arabidopsis thaliana, Solanum tuberosum* and other species indicated that all the SlPIP2s sequences belonged to the MIP protein superfamily. As shown in Fig. 2, SlPIP2;1, SlPIP2;7 and SlPIP2;5 were all clustered with PIP2, 37 plasma membrane aquaporins were split into two groups which could be further divided into two subgroups.

**Figure 3:**
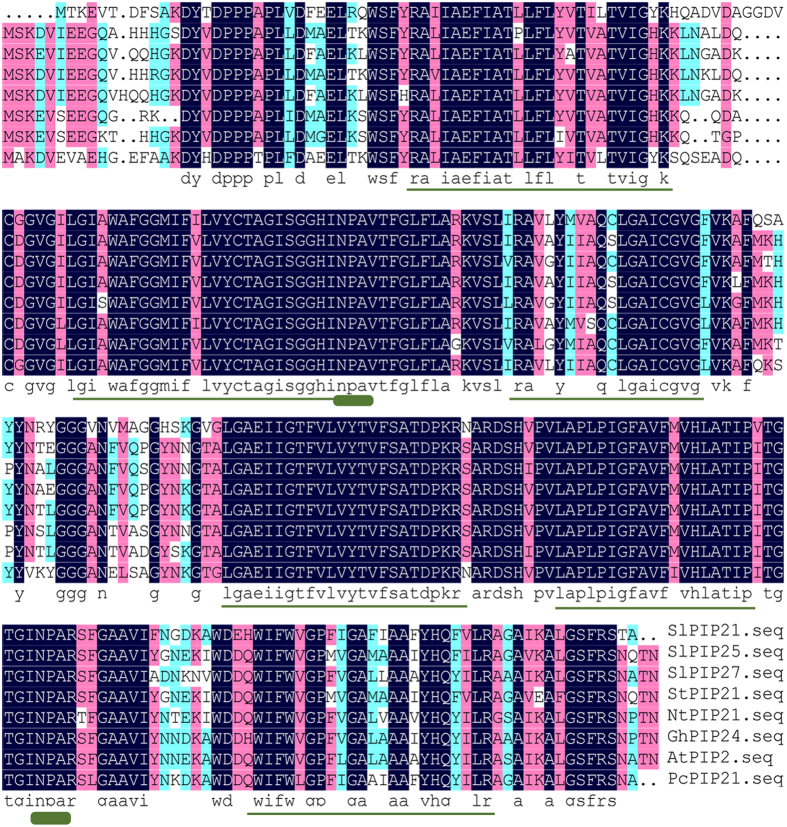
Alignment of predicted amino acid sequence of tomato aquaporins (*SlPIP2;1, SlPIP2;7* and *SlPIP2;5*) with other aquaporins (DNAMAN). The predicted amino acid sequences of *SlPIP2;1, SlPIP2;7* and *SlPIP2;5* were compared with aquaporins from different sources. Transmembrane domains are shown with a dashed line below the alignment; circles indicate the NPA selectivity filter.

A more detailed analysis concerning the amino acid sequences was performed by multiple alignments. According to BLASTP analysis of sequence identity, the nucleotide sequence highest similarities were 83% (SlPIP2;1 and AtPIP2:AAB36949.1),90% (SlPIP2;7 and StPIP2;1:ABC01884.1) and 88%(SlPIP2;5 and NtPIP2;1:AAL33586.1), respectively (Fig. 3). The results of multiple alignments indicated that the structure and the amino acid sequences of PIP2 proteins are highly conserved.

SlPIP2;1, SlPIP2;7 and SlPIP2;5 all contained six alpha-helical transmembrane domains and five inter-helical loops, and also include two highly conserved NPA (Asn-Pro-Ala) and the ar/R (Phe-His-Thr-Arg) motifs. Both N-terminus and C-terminus of the PIP2s are less conserved.

### Subcellular localization of SlPIPs

**Figure 4:**
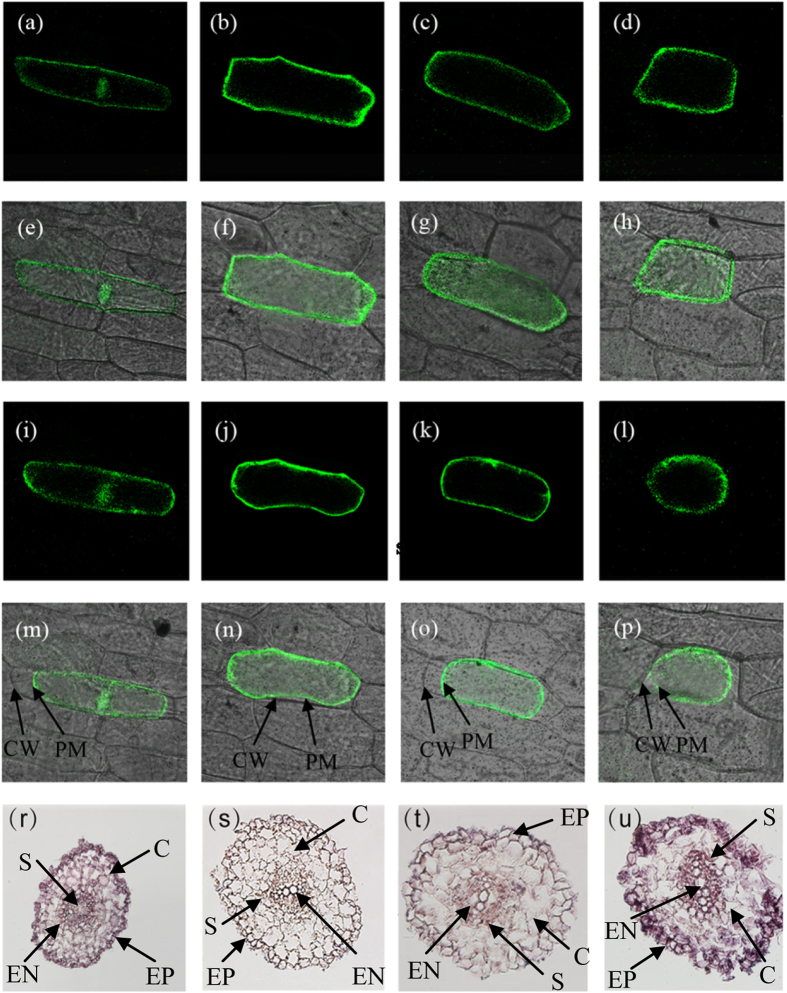
(**a**–**p**) Subcellular localizations of SlPIP2;1, SlPIP2;7 and SlPIP2;5 in onion epidermal cells. The fusion protein SlPIP2;1–GFP (**a**,**e**,**i**,**m**), SlPIP2;7–GFP (**b**,**f**,**j**,**n**), SlPIP2;5–GFP (**c**,**g**,**k**,**o**) and GFP alone (**d**,**h**,**l**,**p**) were transiently expressed under the control of the cauliflower mosaic virus 35S promoter in onion epidermal cells.Images are dark field for green fluorescence (**a**–**d**, **i**–**l**,); merged (**e–h**, **m**–**p**); plasmolysed the cells with 0.8 M mannitol (**i–p**). PM, plasma membrane; CW, cell wall. (**r**–**u**) Tissue localization of expression of aquaporins in roots of tomato. Expression was analyzed by *in situ* hybridization in root. Three candidate aquaporin genes SlPIP2;7 (**t**) and SlPIP2;1 (**u**) were studied, panel (**r**) is the positive control and panel (**s**) is the negative control. EP:epidermis, C: cortex, EN: endodermis, S: stele. Bar = 400 μm.

The subcellular localizations of SlPIP2;1-GFP, SlPIP2;5-GFP and SlPIP2;7-GFP fusion proteins were determined in onion epidermal cells using fluorescent microscopy. As shown in Fig. 4(a–p), the onion epidermal cells transformed with only the p35S: GFP vector exhibited fluorescence throughout the cells. By contrast, cells transiently expressing SlPIP2;1-GFP, SlPIP2;5-GFP and SlPIP2;7-GFP all displayed sharp fluorescent signals at the outer layer. To further distinguish localization in the plasma membrane or in the cell wall, cell plasmolysis was induced by adding 0.8 M mannitol. In the plasmolysed cells, the fluorescence of SlPIP::GFP was observed exclusively in the plasma membrane, indicating that *SlPIP2;1, SlPIP2;5* and *SlPIP2;7* encode plasma membrane-located proteins and these three tomato aquaporin genes all belong to SlPIP family.

### Tissue localization of expression

Since these three aquaporins (*SlPIP2;1, SlPIP2;5* and *SlPIP2;7*) are highly expressed in roots, they were selected for an analysis of their tissue localization of expression using *in situ* hybridization (Fig. 4(r–u)). The result revealed two distinct expression patterns. *SlPIP2;7* (Fig. 4t) and *SlPIP2;5* (data did not shown) expression was localized mainly in the epidermis and stele, with comparatively little expression in the cortex and endodermis. The tissue expression pattern of *SlPIP2;1* (Fig. 4u) differed from that of the other aquaporins, and was expressed almost ubiquitously in all major root tissues, including epidermis, cortex, endodermis, and stele.

### Functional analysis of SlPIP2s in ***X. laevis oocytes.***

**Figure 5:**
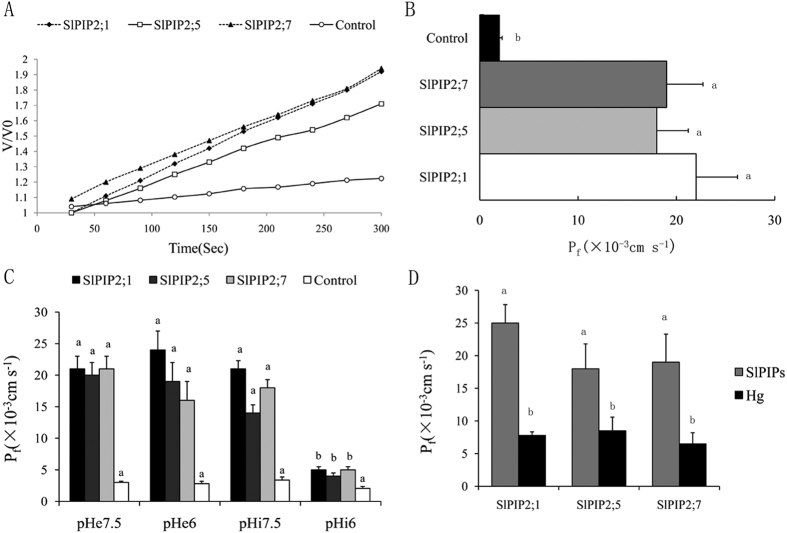
Assay on functional expression of *SlPIP2;1, SlPIP2;7* and *SlPIP2;5* in *Xenopus* oocytes. (**A**) Increase in relative volume of oocytes injected with *SlPIP2;1, SlPIP2;7* and *SlPIP2;5* cDNA after transfer to hypoosmotic medium, using water as control. (**B**) Pf-values of oocytes injected with *SlPIP2;1, SlPIP2;7* and *SlPIP2;5* cRNA, using water as the control. Pf of oocytes expressing *SlPIP2;1, SlPIP2;7* and *SlPIP2;5* present significant differences from negative control. Significant differences were determined by Duncan’s multiple range test (P < 0.05). Error bars indicate SD (n = 3). (**C**) *SlPIP2;1, SlPIP2;7* and *SlPIP2;5* expressing oocytes were exposed to different external (pHe) or internal (pHi) pH conditions. Pf for oocytes expressing SlPIP2s exposed to internal acidification is statistically different from itscontrol (i.e. pHi = 7.5), while treatment with pHe = 6.0 does notresult in a significant inhibition when compared with itscontrol (pHe = 7.5). Significant differences were determined by Duncan’s multiple range test (P < 0.05). Error bars indicate SD (n = 3). (**D**) *SlPIP2;1, SlPIP2;7* and *SlPIP2;5* expressing oocytes were exposed to 100 mM HgCl_2_ condition. Pf for oocytes expressing SlPIP2s exposed toHg result in a significant inhibition when compared with itscontrol. Significant differences were determined by Duncan’s multiple range test (P < 0.05). Error bars indicate SD (n = 3).

In order to elucidate whether SlPIP2s expression enhances water permeability, capped sense cRNAs of SlPIP2 genes were injected into *X. laevis oocytes*. After incubation for 2 days for cRNA translation and protein localization to cell membranes, *Xenopus* oocytes expressing SlPIP2s were exposed to a hypo-osmotic shock of 160 mOsmkg. The resulting increase in volume was calculated by measuring the increase in area over time. From the increase in oocyte volume plotted versus time, water permeability was calculated and compared with that of water-injected oocyte. The experimental results are presented in (Fig. 5A,B). As expected, expression of SlPIP2;5 in *Xenopus* oocytes led to an almost 8-fold increase of the swelling rate compared with the water-injected oocytes (control). The oocytes expressing SlPIP2;1 and SlPIP2;7 protein showed Pf of 21.9 ± 4.32 10^−8^ cm/s (n = 20) and 19.3 ± 3.89 10^−8^ cm/s (n = 20) respectively. which was about 10-fold higher than that of the controls (2.13 ± 0.28 10^−8^ cm/s, n = 20). From the calculated Pf values, SlPIP2;1, SlPIP2;7 and SlPIP2;5 can be characterized as water channels with high water permeability.

As we know, aquaporins are sensitive to Hg and pH. In order to test if SlPIP2s also show a functional blockage of activity, oocytes expressing SlPIP2s were exposed to different pH values and 100 mM HgCl_2_. The results (Fig. 5C) confirm that SlPIP2s partially shut down water permeability when the oocyte internal pH was acidified. By contrast, external pH acidification did not affect SlPIP2 activity. The Hg inhibitory response on SlPIP2s was also tested and, in this condition the inhibition of SlPIP2;1, SlPIP2;5 and SlPIP2;7 was 64%, 52% and 58%, respectively (Fig. 5D). The above results clearly demonstrate that SlPIP2s are highly inhibited by Hg and low internal pH.

### Root hydraulic conductivity of tomato

**Figure 6:**
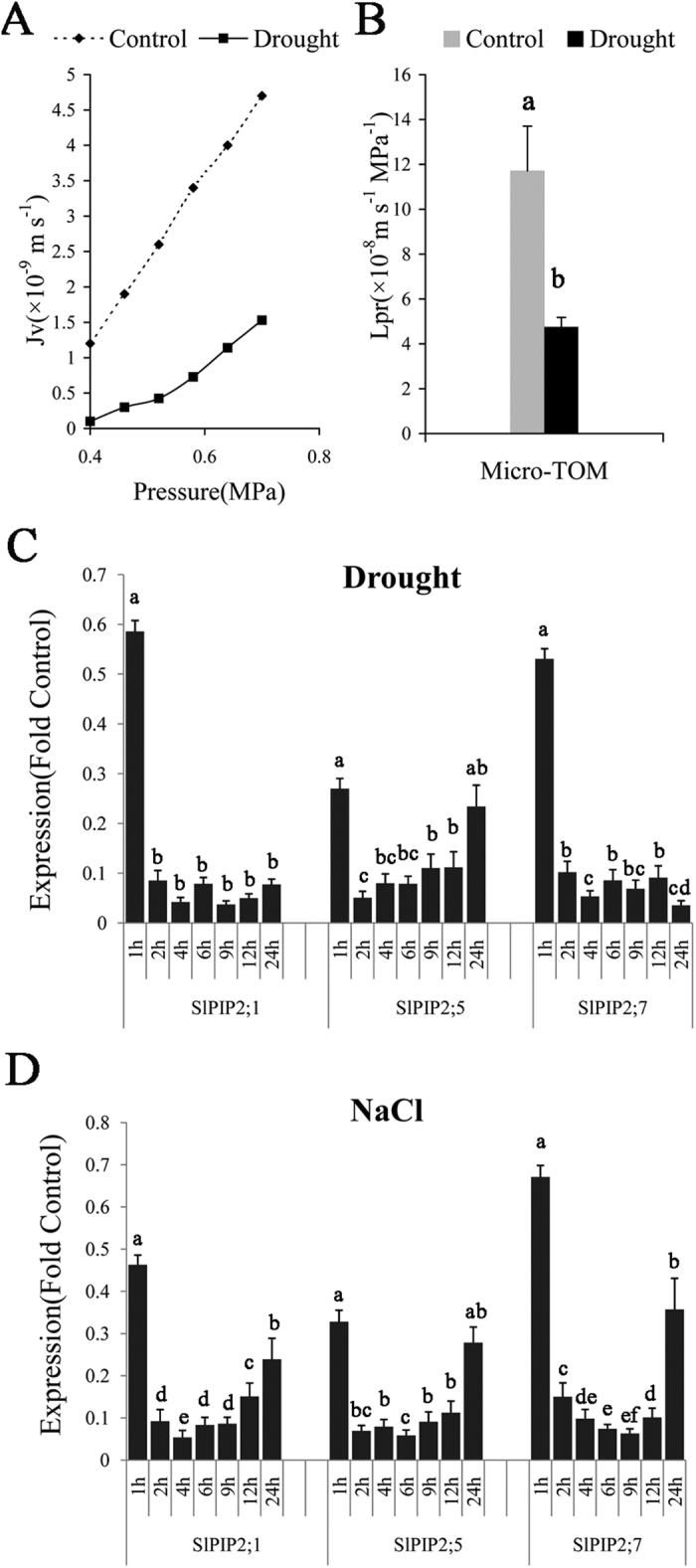
Root hydraulic conductivity of tomato plants. (**A**) The relation between P to Jv of water supplied in tomato root system. (**B**) Hydraulic conductivity of tomato roots under normal and drought stress. Theroot hydraulic conductance was down regulated under drought treatment. Significant differences were determined by Duncan’s multiple range test (P < 0.05). Error bars indicate SD (n = 3). Drought (**C**) and salt (**D**) regulation of *SlPIP2;1, SlPIP2;7* and *SlPIP2;5* expression in tomato roots. Significant differences were determined by Duncan’s multiple range test (P < 0.05). Error bars indicate SD (n = 3).

Osmotic Lp_r_ can be considered an index of water flow via the cell-to-cell pathway, and it is strongly dependent on the density or activity of water channels in the plasma membranes of root cells^8^. To understand the effects of water deficiency on root hydraulic conductivity, we applied 10%PEG6000 on tomato roots and measured the osmotic root hydraulic conductance (Lp_r_) and sap flow rate (Jv) after 2 h. The results demonstrate plants maintained a 2.4-fold lower Lp_r_ (Fig. 6A; Lp_r_ 4.755 × 10^−8^ m s^−1^ MPa^−1^)compared with the untreated controls(Fig. 6B; Lp_r_ 11.714 × 10^−8^ m s^−1^ MPa^−1^) under drought treatment.

### Expression levels of the three SlPIPs genes are regulated in roots upon abiotic stress

In order to test whether the mid-to-long-term (greater than 2 h) inhibition of Lp_r_ by drought is accompanied by a reduced *SlPIP*s transcripts, total RNA were exacted from roots under drought treatment from 0 h to 24 h. The expression profile of the three genes are shown in (Fig. 6C,D). The expression levels in roots sharply decreased after 1 h, and remained down regulated from 2 to12 h, with a slight increase after 24 h. Previous studies in *A. thaliana* have shown that salty environments may also induce a reduction of aquaporin transcript levels. A similar result was observed in salt-treated tomato seedlings. The transcripts of *SlPIP2;1, SlPIP2;7, SlPIP2;5* decreased dramatically after 1 h and reached minimum levels at 4 h, 9 h, 2 h, respectively, and increased slightly after 24 h treatment. These results suggest that three SlPIPs participate in the response to both salt and drought stress.

### Overexpression of SlPIP2;1, SlPIP2;7 and SlPIP2;5 enhanced tolerances to drought stress

**Figure 7:**
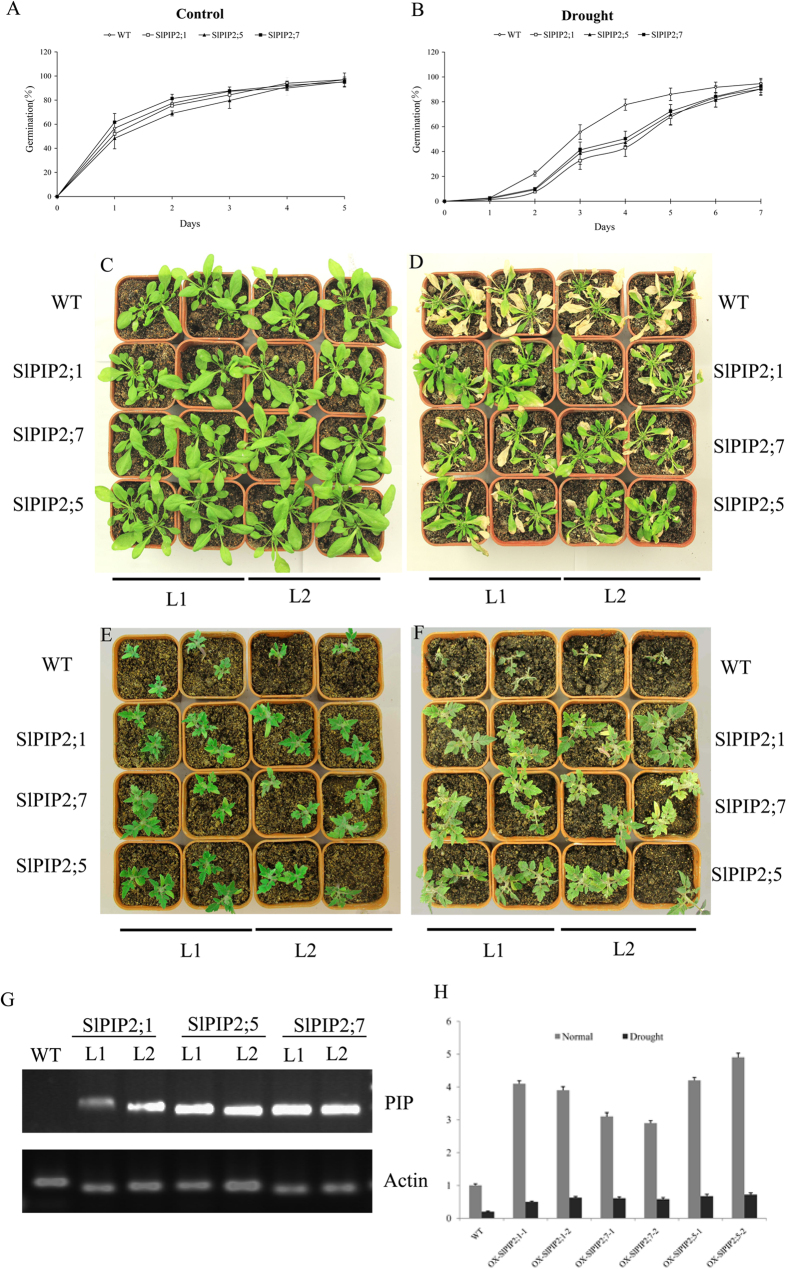
Growth of the wild-type and transgenic plants under normal and drought growth conditions. (**A**) Germination rates of the wild-type and transgenic Arabidopsis under normal conditions. Error bars indicate SD (n = 3). (**B**) Germination rates of the wild-type and transgenic Arabidopsis under drought conditions. Error bars indicate SD (n = 3). (**C**) The wild-type and transgenic Arabidopsis plants were photographed 20 days after germination on soil before drought treatment. (**D**) Phenotypes of wild-type and transgenic Arabidopsis plants subjected to drought stress for 10 days. (**E**) The wild-type and transgenic tomato plants were photographed 30 d after germination on soil before drought treatment. (**F**) Phenotypes of wild-type and transgenic tomato plants subjected to drought stress for 15 days. L1: Transgenic line1; L2: Transgenic line2.Confirmation of the transgenic lines. RT- PCR or real time quantitative analyses of the expression of SlPIP2;4, SlPIP2;5 or SlPIP2;7 in the wild-type plants and independent transgenic lines (L-1and L-2) in (**G**) two week-old whole Arabidopsis plants grown in MS medium and (**H**) two-week-old tomato plants grown in MS medium.

To examine the response of transgenic plants overexpressing a single aquaporin isoform under drought stress condition, transgenic Arabidopsis and tomato lines constitutively overexpressing *SlPIP2;1, SlPIP2;7* or *SlPIP2;5* were generated. The expression of SlPIP2; 1, SlPIP2; 7 and SlPIP2; 5 in T3 transgenic Arabidopsis plants was confirmed by RT-PCR analysis (Fig. 7G). We also examined the expression of SlPIP2;1, SlPIP2;7 and SlPIP2;5 in T2 transgenic tomato plants by Real-Time PCR both under normal and the drought condition (Fig. 7H). Transgenic Arabidopsis and tomato plants were used for germination and growth comparison under both regular and drought stress conditions. There were no obvious differences in germination between the wild type and three transgenic *Arabidopsis* lines (35S::SlPIP2; 1, 35S::SlPIP2;7 and 35S::SlPIP2;5) under the well-watered condition (Fig. 7A). In contrast, when the seeds of wild type and 35S::SlPIP2;1, 35S::SlPIP2;7, 35S::SlPIP2;5 *Arabidopsis* plants were germinated in MS with PEG6000, about 50% of transgenic seeds germinated at day 3, while only 32% of the wild type seeds germinated. On the seventh day, the germination rates of 35S::SlPIP2;1, 35S::SlPIP2;5 and 35S::SlPIP2;7 seeds were 92%, 91% and 93%, while only 84% of wild type seeds germinated (Fig. 7B). Similar patterns of retarded germination were also observed for the transgenic tomato plants compared with wild type plants under dehydration stress (data not shown).

To further confirm the effect of 35S::SlPIP2;1, 35S::SlPIP2;7 and 35S::SlPIP2;5 overexpression on the growth performance of *Arabidopsis* under drought stress, water was with held from 3-week-old *Arabidopsis* plants. The non-transformed control plants started wilting 10 days after the termination of irrigation, at which time the transgenic *Arabidopsis* plants still showed a nearly normal phenotype (Fig. 7C,D). In addition, these three genes (*SlPIP2;1, SlPIP2;7, SlPIP2;5*) were contributed to the stress resistance of plant under drought condition, as demonstrated in both Arabidopsis and tomato. In the test of drought stress tolerance, after 15 d, almost all the SlPIP2 overexpression tomato plants still grew well, while the wild type plants were seriously damaged (Fig. 7E,F). In our work, 63%, 56% and 62% of SlPIP2;1, SlPIP2;7, SlPIP2;5 overexpression tomato plants survived, respectively, However, for the wild-type plants, survival rate was only 47% (Fig. S2).

Since it is apparent that overexpression of *SlPIP2;1, SlPIP2;7* and *SlPIP2;5* in *Arabidopsis* plants increased survival rates of the plants under drought stress, we next analyzed root hydraulic conductance (Lp_r_) to investigate whether these changes in phenotype are directly related to the water transport activities of aquaporins under regular and drought stress conditions. No significant difference was found between the Lp_r_ of transgenic plants and that of wild-type plants under normal conditions. However, after being subjected to water deficit, transgenic plants showed higher Lp_r_ than that of wild type plants (Table 1).

**Figure 8:**
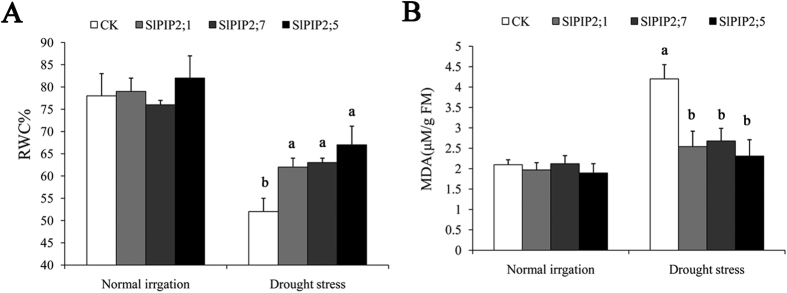
Analysis of RWC and MDA in transgenic lines under drought stress. Tomato leaves were sampled from WT and transgenic lines under drought stress for 15 d to detect RWC (**A**) and MDA (**B**). Significant differences were determined by Duncan’s multiple range test (P < 0.05). Error bars indicate SD (n = 3).

Transgenic tomato plants were employed for the measurements of malondialdehyde (MDA) and relative water content (RWC). After 15 d drought stress, the WT displayed greater decrease in RWC than transgenic plants (Fig. 8A). In addition, MDA (Fig. 8B) was lower, showing that under drought condition, the three groups of transgenic plants are equally capable of enduring debilitating stress conditions in a better way than the untransformed control plants.

## Discussion

### SlPIP2;1, SlPIP2;7 and SlPIP2;5 encode water channels with high water permeability (Pf)

To our knowledge, *SlPIP2;5* is a highly expressed PIP2 aquaporin described in roots in addition to genes *SlPIP2;7* and *SlPIP2;1*. Topology and hydrophobicity predictions using TMHMM program revealed that the SlPIP2 proteins contain the MIP family signature sequence HVNPAVTFG and two special “NPA” motifs, fitting the pattern characteristic of other members of the aquaporin super family. In addition, they also constitute six transmembrane helixes and five loops, similar to the known PIP members. Phylogenetic analysis of full-length AQP cDNA clones showed that SlPIP2s have a closer evolutionary relationship with PIP2s (86–90% identity) than that of another PIP1 subgroup (70–82% identity). Additionally, fluorescence microscopy revealed that recombinant SlPIP2s::GFP are produced and transported to the plasma membrane in onion epidermal cells. Taken together, these data strongly indicate that the isolated SlPIP2s genes belong to the PIP2 subgroup.

SlPIP2;1, SlPIP2;7 and SlPIP2;5 are water channels which promote very high membrane Pf, and these aquaporins seem to be mainly involved in water transport. *Arabidopsis* RD28 was identified with high water channel activity which increased the osmotic permeability of the oocytes by 10- to 30-fold^44^. TdPIP2;1, as a PIP2 member in wheat, also possesses high water transport activity^45^. In cotton, expression of GhPIP2;3 also increased a high permeability coefficient of oocytes^46^. Similarly, tomato SlPIP2;1, SlPIP2;7 and SlPIP2;5 proteins displayed functional water channel activity which increased the osmotic permeability of the *X. laevis* oocytes 8- to 10-fold (Fig. 5B), demonstrating that they are also functional PIP proteins in tomato.

The mercury sensitivity in *Beta vulgaris*, BvAQP is attributed to binding to Cys-189 and Cys-181, respectively with both residues located in extracellular loop E and in close proximity to the pore^47^. Interestingly, Bienert *et al*. showed that the loop A cysteine of ZmPIP1;2 is involved in the mercury sensitivity of the channels^48^. A similar result was found in SlPIP2s; incubation of SlPIP2-injected oocytes in 100 mM HgCl_2_ reduced water permeability compared to untreated oocytes, confirming its sensitivity to mercury.

When oocytes expressing SlPIP2s are subjected to cytosolic acidification, membrane Pf is partially shut down (Fig. 5C). These results are in accordance with the presence of the highly conserved His199 in the PIP2 sequence, a residue shown to be responsible for water transport blockage under cytosolic acidification in other PIP2 aquaporins^49^. A similar response was observed in *Beta vulgaris*^50^ where the Pf was significantly reduced in response to cytosolic acidification. In *Fragaria ananassa*, the reduction of water transport under cytosolic acidification detected for FaPIP2;1 (partial inhibition) compared with FaPIP2;1-FaPIP1;1 co-expression (total inhibition)^51^. Regulation of plant aquaporin conductivity was suggested to be achieved by a gating mechanism that involves protein phosphorylation under drought stress conditions and protonation after cytosolic acidification during flooding^52^,^53^.

### Root hydraulic conductivity and expression of SlPIP2;1, SlPIP2;7 and SlPIP2;5 under abiotic stress in tomato

Although in some reports, drought caused an increase in hydraulic conductivity (Lp_r_)^17^,^54^, most Lp_r_ decreases upon root exposure to drought^16^. For *Agave deserti* roots, Lp_r_ decreased by 30–60% during 10 d in drying soil and after soil rewetting, Lp_r_ increased to pre-drying values^55^. Compared with non-water stress, root hydraulic conductivity of intact roots was decreased by 29% in *Oryza sativa* L. Gao *et al*.,^56^. The role of water channel proteins (aquaporins) and their regulation as related to hydraulic conductivity have been the subject of intense research^13^. Based on measurements of osmotic water permeability of isolated membranes^57^, PIPs, in particular PIP2s are the most likely candidates limiting cell hydraulic conductivity. Expressions of three SlPIP2s genes were also markedly downregulated by water deficiency in the root, while the hydraulic conductivity of the roots decreased. This result is consistent with previous study. Compared to the wild-type plant, the overexpressing aquaporin RWC3 transgenic lowland rice RWC3 exhibited higher root osmotic hydraulic conductivity (Lp) under drought treatment^35^.

Following drought and salt stress treatments, the expression of three tomato PIP genes was down-regulated (Fig. 6C,D), indicating that *SlPIP2;1, SlPIP2;7* and *SlPIP2;5* are sensitive to high concentration of salt or PEG treatment. Nguyen *et al*. showed that 3 rice aquaporin genes were markedly downregulated by water deficiency in the root, suggesting that these candidate genes are key regulators of water uptake from the soil^32^. Two *Fragaria vesca* aquaporins highly expressed in the root under normal conditions were down-regulated in the root correlative to the level of drought stress^58^. Similarly, the expression of *SlPIP2;1, SlPIP2;7* and *SlPIP2;5* was down-regulated in roots by drought and salt treatment. In brief, our results revealed that the isolated tomato PIPs *SlPIP2;1, SlPIP2;7* and *SlPIP2;5* may play significant roles in regulating osmotic balance of root cells when plants encounter environmental stresses. Jang found that the expression levels of most AtPIPs were markedly down-regulated in the roots by drought treatment, while they were highly up-regulated in salt-treated roots^59^. Increased AQP levels might provide the plant with an additional capacity to cope with a water deficit, based on observations that some AQPs are induced or activated at the onset of drought^60^. These results show that transcription of PIP aquaporins can be fine-tuned with the environment in response to declining water availability.

### Overexpression SlPIP2;1, SlPIP2;7 and SlPIP2;5 can enhance plant abiotic stress tolerance

Aquaporins function in rapid transmembrane water flow during growth and development and play important roles in maintaining plant water relations under drought conditions. Previous studies have shown that SlTIP2;2 in tomato increased the osmotic water permeability of the cell and whole-plant transpiration^36^. In our study, we observed that expression of SlPIP2;1, SlPIP2;5 and SlPIP2;7 in roots were deduced after drought and salt treatment, implying that these genes product may play a significant role in mediating response to drought and salt stresses. To better understand the function of SlPIP2s during abiotic stress, we overexpressed three SlPIP2s genes in *Arabidopsis* and tomato. Compared with controls, the *Arabidopsis* plants overexpressing *SlPIP2;1, SlPIP2;7* and *SlPIP2;5* showed higher hydraulic conductivity levels, germination and survival rates under both normal and water-stress conditions. Similar results were also reported in cotton and tobacco transgenic plants^61^,^62^. In contrast to controls, the expression of NtAQP1 in tomato plants resulted in improved water use efficiency and hydraulic conductivity under abiotic stress^63^. Although our results are consistent with many studies, some researchers propose that overexpression of PIP type aquaporin enhances plant wilting in response to drought stress^6^,^63^. Currently, there are two opposing views concerning the mechanism by which this occurs. One theory hypothesizes that increased AQP levels might provide the plant with an additional capacity to cope with a water deficit. Another opinion is that plants may avoid an excessive loss of water by down-regulating AQPs during dehydration^64^. In our case, the results supported the first opinion.

We observed that SlPIP2s -overexpressing plants exhibited better growth compared to WT plants under drought conditions, indicating a positive influence of SlPIP2s on water retention. Consequently, we investigated the physiological mechanisms involved in improved water retention conferred by SlPIP2s. SlPIP2s -overexpressing transgenic plants maintained lower level of MDA compared to WT plants subjected to similar drought treatment. At the same time, the hydraulic conductivity (Lpr) of transgenic plants appeared differently under normal conditions and drought stress. Root Lpr, in transgenic plants did not differ from that in control plants under normal irrigation. However, the transgenic plants decreased their Lpr only by about 24% under drought stress, while Lpr of control plants decreased more than 43%. This result indicates thatSlPIP2s may function in maintaining osmotic adjustment under drought stress. To our knowledge, this study is the first to demonstrate that SlPIP2;1, SlPIP2;7 and SlPIP2;5 are key genes that can be utilized to improve water use efficiency and enhance plant tolerance under drought stress.

To better understand the regulatory mechanisms of SlPIP2s regulation abiotic stress tolerance, we suppressed (RNA interference, RNAi) SlPIP2s-RNAi in tomato. RNAi strongly down-regulated SlPIP2;7 by 26–42% of the wt level, while the level of SlPIP2;1 and SlPIP2;5 were down-regulated by 37–43% and 40–50% of the wt level, respectively (Fig. S3). Unfortunately, SlPIP2s-RNAi plants showed no changes in water status, same phenotype under both normal and drought condition. Similar result was also found in Arabidopsis^26^.

## Conclusion

In conclusion, this work provided novel information to increase knowledge about the response of transgenic plants overexpressing a single aquaporin to drought condition in *Arabidopsis* and tomato. *SlPIP2;1, SlPIP2;7* and *SlPIP2;5* were identified as candidate aquaporins based on their significantly high expression in roots. In addition, the inhibition of root hydraulic conductivity by drought is accompanied by a reduced of SlPIPs transcripts. The overexpression of SlPIP2;1, SlPIP2;7 and SlPIP2;5 transgenic Arabidopsis and tomato plants showed better water status and survival rates than wild type plants under drought stress condition. The present results emphasize the importance of aquaporin-mediated water transport in response to drought stress in tomato plant^65^.

## Additional Information

**How to cite this article**: Li, R. *et al*. Plasma Membrane Intrinsic Proteins *SlPIP2;1, SlPIP2;7* and *SlPIP2;5* Conferring Enhanced Drought Stress Tolerance in Tomato. *Sci. Rep.* **6**, 31814; doi: 10.1038/srep31814 (2016).

## Supplementary Material

Supplementary Information

## Figures and Tables

**Table 1:** Root hydraulic conductivity of wild-type and transgenic Arabidopsis plants under normal and drought conditions.

Transgenic Plant	Lp_r_(10^−8^ cm s^−1^ MPa^−1^)	Drought
	Control	
Arabidopsis-Wild Type	5.698 ± 0.197 a	3.245 ± 0.052 b
Arabidopsis-SlPIP2;1	6.264 ± 0.198 a	4.852 ± 0.048 c
Arabidopsis-SlPIP2;5	5.840 ± 0.123 a	4.392 ± 0.013 c
Arabidopsis-SlPIP2;7	5.944 ± 0.147 a	4.504 ± 0.016 c
Tomato-Wild Type	8.922 ± 0.283 a	5.326 ± 0.098 c
Tomato-SlPIP2;1	9.428 ± 0.219 a	6.738 ± 0.082 b
Tomato-SlPIP2;5	10.392 ± 0.201 a	6.983 ± 0.023 b
Tomato-SlPIP2;7	9.873 ± 0.198a	6.562 ± 0.031 b

Significant differences were determined by Duncan’s multiple range test (P < 0.05). Error bars indicate SD (n = 3).
