# Supplementary material for: Plasma Membrane Intrinsic Proteins SlPIP2;1, SlPIP2;7 and SlPIP2;5 Conferring Enhanced Drought Stress Tolerance in Tomato
Source: Sci Rep. 2016 Aug 22;6:31814. doi: 10.1038/srep31814 (PMC4992886; doi:10.1038/srep31814)
Supplement: Supplementary Information [file srep31814-s1.doc]

**Plasma Membrane Intrinsic Proteins *SlPIP2;1, SlPIP2;7* and *SlPIP2;5***

**Conferring Enhanced Drought Stress Tolerance in Tomato**

Ren Li^1, 2^, Jinfang Wang^1^, Shuangtao Li^1^, Lei Zhang^1^, Chuandong Qi^1^, Sarah Weeda^3^, Bing Zhao^1^*, Shuxin Ren^3^, Yang-Dong Guo^1^*

*^1^ College of Horticulture, China Agricultural University, 100193 Beijing, China*

*^2^Institute of Vegetables and Flowers, Chinese Academy of Agricultural Sciences, 100081Beijing, China*

*^3^ School of Agriculture, Virginia State University, PO Box 9061, Petersburg, VA 23806, USA.*

Table S1. Gene names, accession numbers, and the length of deduced polypeptides of 11 PIP and 9 TIP tomato aquaporin genes.

| **Gene names** | **Accession numbers** | **Length of deduced polypeptides** |
| --- | --- | --- |
| SlPIP21 | Solyc06g011350.2.1 | 280 |
| SlPIP22 | Solyc10g084120.1.1 | 281 |
| SlPIP24 | Solyc11g069430.1.1 | 287 |
| SlPIP25 | Solyc02g083510.2.1 | 259 |
| SlPIP27 | Solyc01g111660.2.1 | 283 |
| SlPIP28 | Solyc05g055990.2.1 | 280 |
| SlPIP11 | Solyc08g081190.2.1 | 286 |
| SlPIP12 | Solyc03g096290.2.1 | 286 |
| SlPIP13 | Solyc08g008050.2.1 | 287 |
| SlPIP14 | Solyc12g056220.1.1 | 288 |
| SlPIP15 | Solyc01g103270.2.1 | 283 |
| SlTIP11 | Solyc10g083880.1.1 | 247 |
| SlTIP21 | Solyc12g044330.1.1 | 248 |
| SlTIP32 | Solyc03g019820.2.1 | 260 |
| SlTIP | Solyc06g060760.2.1 | 250 |
| SlTIP22 | Solyc06g066560.1.1 | 273 |
| SlTIP13 | Solyc06g075650.2.1 | 253 |
| SlTIP14 | Solyc08g066840.2.1 | 247 |
| SlTIP15 | Solyc06g074820.2.1 | 250 |
| SlTIP12 | Solyc03g120470.2.1 | 276 |

Table S2. Primers for quantitative RT-PCR analysis

| **Gene names** | **Primer** |
| --- | --- |
| SlPIP21 | F：ACGTACCCGTGTTGGCACCTCTTCC  R：ATGTTCGTCCCACGCCTTGTCACC |
| SlPIP22 | F：ATTGACCCTGAGGAACTTGGAAAA  R：TCACCATCACTTTGGCTCTTGTAG |
| SlPIP24 | F：TACTCCGCAAAGGATTACACTGAT  R：AGCCCAAGCAATACCAAGTAAACC |
| SlPIP25 | F：GTCCTCTTCCAGCCATCCA  R：ACCACTGAGCACAATGTTACCG |
| SlPIP27 | F：ATTCCCATATCCCTGTGTTGGCTCC  R：AGCTGCAGCTCTCAAAATGTATTGG |
| SlPIP28 | F：ATACCCAACGTGTAGCATCACTCTC  R：CCAGCAGTGGAATACACGAGAACA |
| SlPIP11 | F：CTATCATCTACAACGACGAGCA  R：CATTGAAGGAGAAACTTGAACA |
| SlPIP12 | F：TTTCACTCACTAACTCCCATCAAT  R：TAAAGAAAGAGGAAAGTAGCCACA |
| SlPIP13 | F：ACCATCAAATAATCATCAGAGCA  R：AGGATAAAATAAAAATTATTTTCAT |
| SlPIP14 | F：GGTGTTGTGAAGGGTTTTATGGTT  R：ACCCAGAAAATCCAGTGGTCATCC |
| SlPIP15 | F：ATGATTATGCCAAGGGAGATGA  R：GCCAAATGAACCAAGAACACAG |
| SlTIP11 | F：CTATTCGTAGCGGTTTCGGTTG  R：TTGTTCCCAAACTACCCTTCTT |
| SlTIP21 | F：TGACTGGAGGAATGGCGGTT  R：ACCACAGCGGGTCCAAATGA |
| SlTIP32 | F：GCTGATTTATTGGTGTTATGGCTATG  R：AGCAAGAACAGAGCCTTCACCG |
| SlTIP | F：AATGGTGAAGATTGCCTTTGGTAG  R：TCAAATGTCCACCTGAGATGTTAG |
| SlTIP22 | F：TTTATCTCCACCTTGCTTTTCG  R：CGGTAACAAACTTGAGGAGGCA |
| SlTIP13 | F：ATCCATAGCACATGCCTTTGCCCTT  R：CCGATGTTTCCAGTCCACCAGTAG |
| SlTIP14 | F：TTATTCGTAAAATCAGTTTCATCA  R：CAAGCAGCAACAGAAGCAAGTAAT |
| SlTIP15 | F：TCATCACTCCCCAACTTGTGCC  R：AAAGCCATACCAGAACCCTGACCT |
| SlTIP12 | F：GATTCATTCAGCGTTGTCTCTCTT  R：AAACGGCTACGAATAGAGCAAATC |

Table S3. Quantitative assay of PIPs and TIPs relative expression in root, stem, flower, and root tissues of tomato plants.

|  | Flower | Leaf | Stem | Root |
| --- | --- | --- | --- | --- |
| SlPIP2;8 | 2.738576 | 3.838325 | 7.8923 | 11.9076 |
| SlPIP2;7 | 3.865468 | 3.90695 | 6.7632 | 21.9856 |
| SlPIP2;1 | 2.223367 | 7.814774 | 8.7572 | 19.7623 |
| SlPIP1;5 | 9.8753 | 10.8765 | 4.7643 | 7.9835 |
| SlPIP1;1 | 0.05176 | 0.644594 | 1.260038 | 4.83229 |
| SlPIP1;3 | 11.62578 | 16.14928 | 14.60754 | 13.13273 |
| SlPIP1;4 | 0.374628 | 1.560209 | 2.213415 | 5.292041 |
| SlPIP1;2 | 4.666288 | 6.150655 | 4.387392 | 13.32419 |
| SlPIP2;2 | 10.03216 | 16.14222 | 13.22081 | 7.953598 |
| SlPIP2;4 | 5.553154 | 5.908061 | 5.458321 | 4.402962 |
| SlPIP2;5 | 11.32495 | 23.9867 | 7.848843 | 21.8932 |
| SlTIP1;1 | 7.032314 | 15.46707 | 12.79674 | 9.973222 |
| SlTIP2;1 | 2.72458 | 3.561239 | 0.946291 | 10.84878 |
| SlTIP3;2 | 8.251951 | 10.91072 | 11.10366 | 5.891777 |
| SlTIP | 7.342672 | 10.35272 | 6.138269 | 7.990837 |
| SlTIP2;2 | 12.98804 | 12.08827 | 8.949781 | 10.89685 |
| SlTIP1;3 | 6.017797 | 8.4519 | 5.632562 | 11.6884 |
| SlTIP1;4 | 10.11458 | 9.098142 | 9.739761 | 11.13026 |
| SlTIP1;5 | 1.482891 | 3.253416 | 3.19692 | 11.7047 |

Table S4. Primers for *in situ* hybridization

| **Gene names** | **Primer** |
| --- | --- |
| 18S ribosome cDNA | F：TTGCAGAATCCCGTGAACCATCG  R：TTTCAACCACCACTTGCCGC |
| SlPIP21 | F：ACACTGTCTTCTCTGCCACTGA  R：GGCAGTGCTCCTGAATGAACCT |
| SlPIP27 | F：TTCCTCTACGCCACAGTCGCTA  R：GGGTCAGTAGCAGAGAAAACAG |
| SlPIP25 | F：TACGGCAACGAAAAAATCTG  R：TTGGTTTGGTTGCTGCGGAA |

Table S5. Primers for cloning SlPIP2 genes ORF sequence

| **Gene names** | **Primer** |
| --- | --- |
| SlPIP21 | F：ATGACTAAAGAAGTAACAGATT  R：TCAGGCAGTGCTCCTGAATGAAC |
| SlPIP27 | F：ATGTCAAAAGAAGTGATTGAAG  R：TTAATTGGTGGCGTTGCTGCGG |
| SlPIP25 | F：ATGTCGAAGGACGTGATTGAAGAAG  R：TTAGTTGGTTTGGTTGCTGCGGAAT |


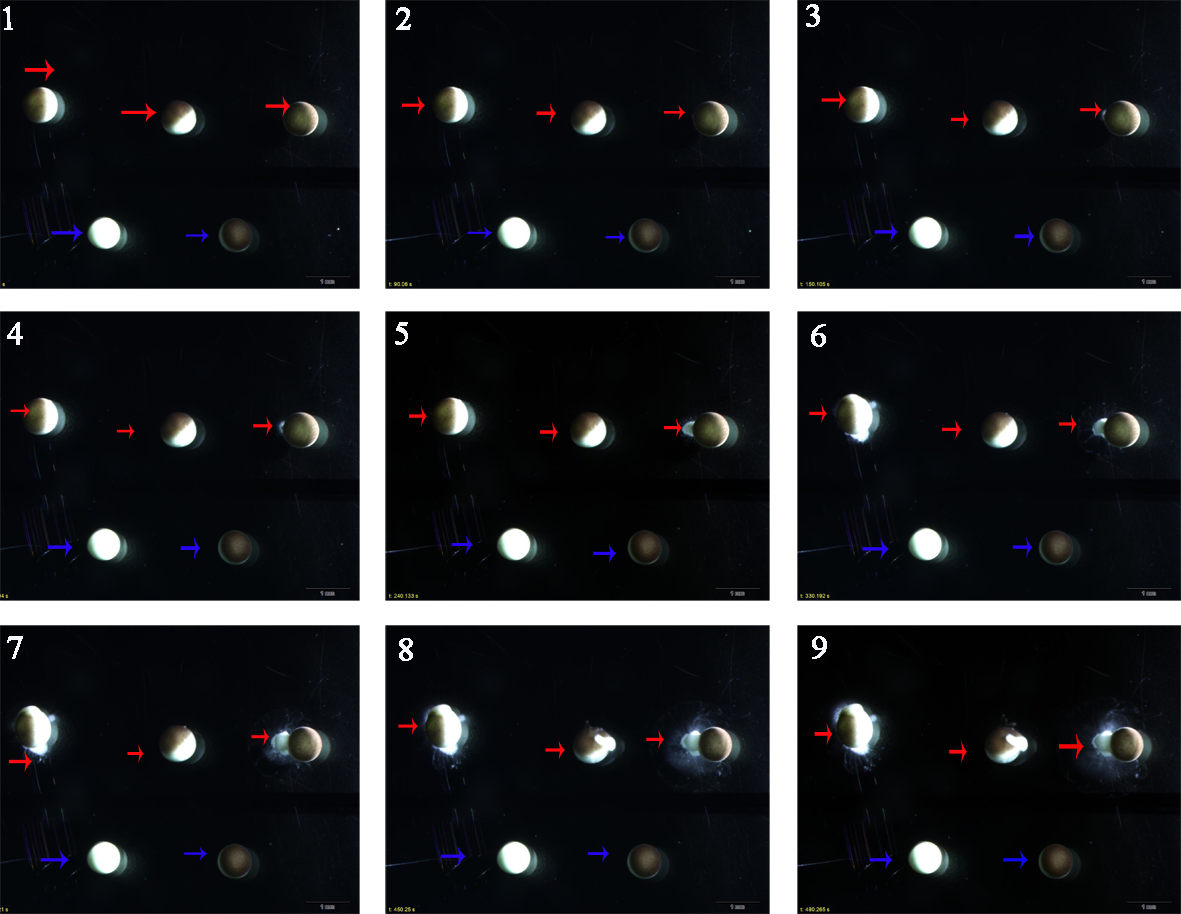


Figure S1. Process of Xenopus oocytes swelling. Red Arrow: injected oocytes with SlPIPs cRNA.

Blue Arrow: negative control.


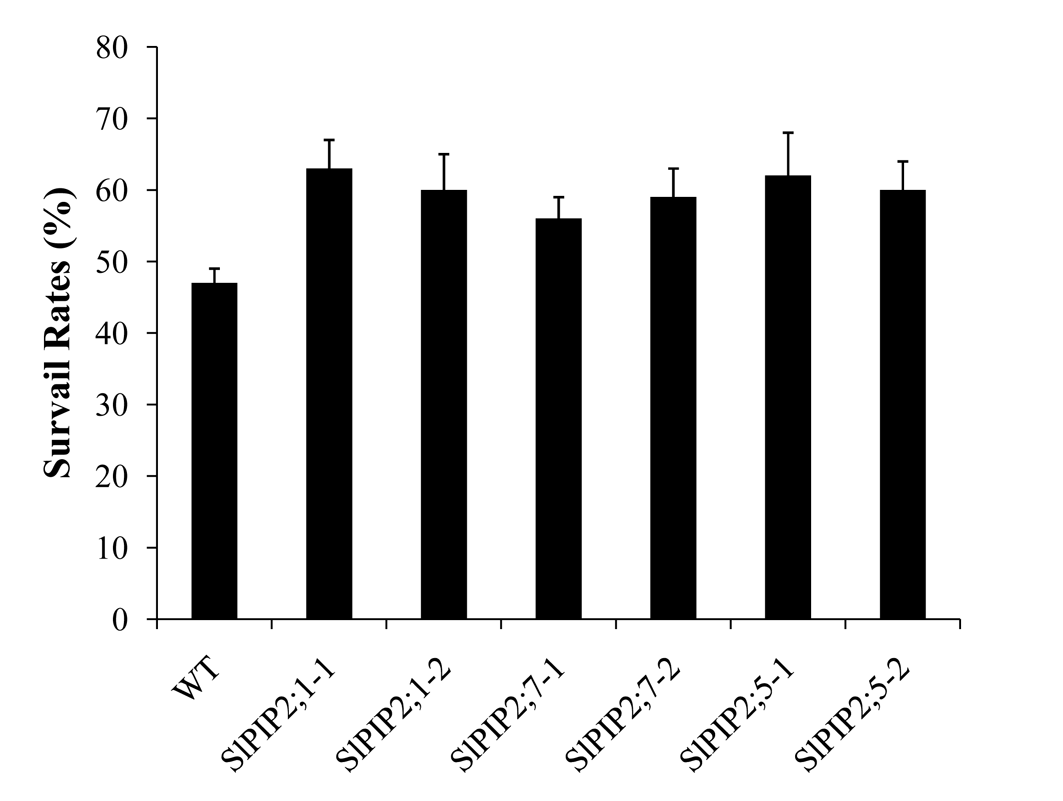


Figure S2. The survival rates of overexpression SlPIP2s lines and WT plants grown under drought stress conditions. Error bars indicate SD (n= 3).


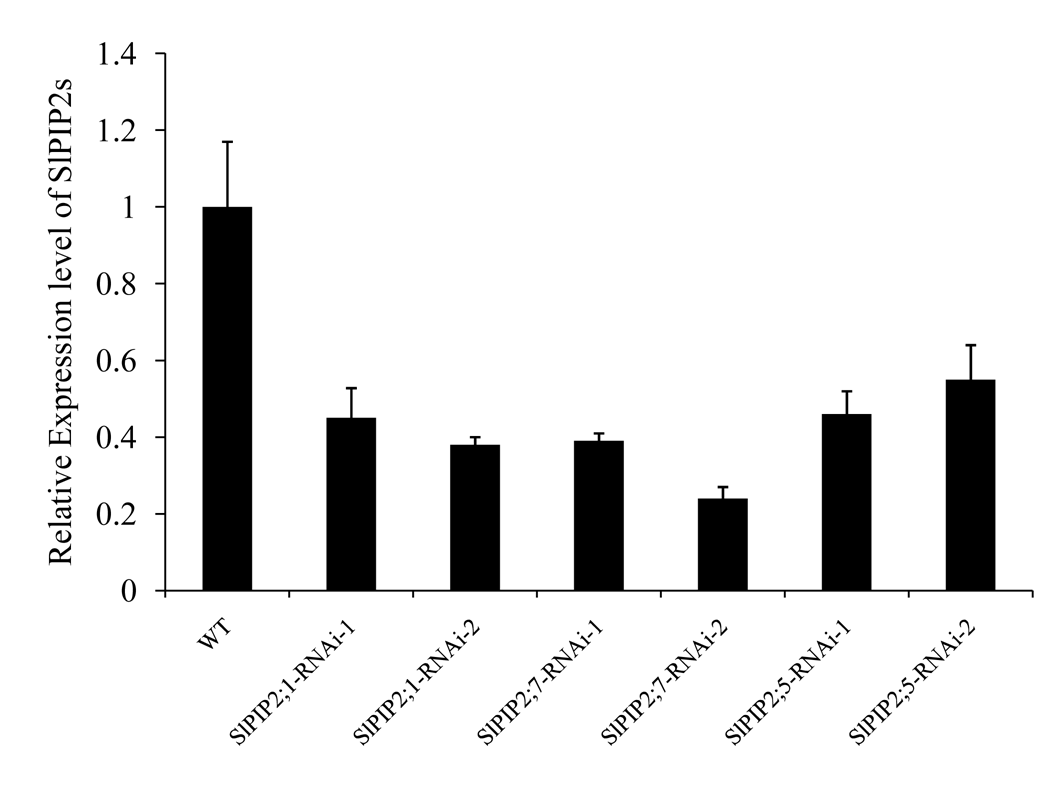


Figure S3. Expression of SlPIP2s in the wild-type and transgenic plants, determined by qRT-PCR.

Error bars indicate SD (n= 3).
